# New compartment model for hepatic blood flow quantification in humans from 15O-water PET images

**DOI:** 10.1007/s00259-025-07210-5

**Published:** 2025-03-21

**Authors:** Oona Rainio, Juhani Knuuti, Riku Klén

**Affiliations:** Turku PET Centre, University of Turku and Turku University Hospital, Turku, Finland

**Keywords:** 15O-water positron emission tomography, Compartmental modelling, Dynamic positron emission tomography, Hepatic blood flow

## Abstract

**Background:**

Different compartment models are commonly used to derive crucial information about blood flow, metabolism, and oxygenation from the results of a dynamic positron emission tomography (PET) scan. However, compared to blood flow in many other organs of interest, the hepatic blood flow (HBF) quantification is challenging due to the dual blood supply of liver from both the hepatic artery and the portal vein (PV). Here, we introduce a new model that can be used to estimate the HBF in combination with an automatic volume of interest selection method.

**Materials and methods:**

By using the $^{15}$O-water PET data of 57 patients, we extract the mean time-activity curves (TACs) from aorta, hepatic PV, liver, and spleen with help of an automated computer tomography-based segmentation tool and systematically fit our new compartment model and three earlier compartment models from literature to the TACs. After this, we compare the model performance with mean relative error (MRE), mean squared error, and Akaike’s information criteria with one-sided Wilcoxon signed-rank tests. After determining the best model, we study possible HBF differences caused by age, sex, and weight with Mann-Whitney U test and Pearson’s correlations coefficient.

**Results:**

We obtained the mean arterial HBF of 0.299±0.168 mL/min/mL, the mean portal HBF of 0.930±0.520 mL/min/mL, and the total HBF of 1.229±0.612 mL/min/mL with our new model. Based on earlier research, both these estimates and also the results of two earlier versions of the original dual-input model are realistic. Out of these three models, our proposed model performed the best in terms of MRE (p-values$\le$0.001). According to our results, there are no significant sex- or age-based differences but there is moderate positive correlation between the arterial and portal HBFs and negative correlation between the total HBF and the weight of patients.

**Conclusion:**

The HBF can effectively be estimated from $^{15}$O-water PET data with our new model in combination with robust segmentation by TotalSegmentator. Due to the fact that potential underestimation of the PV concentration caused by the small size of this vessel might lead to overestimation of the HBF, more research would be beneficial to validate these methods further. Our results suggests that there is a negative trend between the HBF and the weight, though this might be related to the underlying conditions of the patients.

## Introduction

Compartment modeling is a method for extracting relevant information from dynamic positron emission tomography (PET) images with the help of a set of first-order differential equations [2]. A PET image shows the distribution of the given radioactive tracer substance in the human body at a specific moment in time [25] and, in a dynamic image series, each voxel in the three-dimensional space has a time-activity curve (TAC) expressing how the tracer concentration changes with respect to time at this location. By computing a mean TAC of a certain volume of interest (VOI), we can estimate the average tracer concentration within a specific compartment, such as the arterial blood or some organ, and a compartment model is then used to describe the tracer exchange between these compartments [30]. The estimated model parameters tell us about blood perfusion [7], glucose metabolism [10], and tissue oxygenation [28], for instance.

One of the simplest compartment models is the one-tissue compartment model (1TCM). It is generally used for $^{15}$O-water ([$^{15}$O]H$_2$O), a freely diffusable PET tracer containing water labeled with the short-lived radioactive isotope $^{15}$O of oxygen [12]. The fitted 1TCM typically describes the $^{15}$O-water exchange from the arterial blood into the tissue of a single organ or anatomic structure of interest and its exit from this tissue. While the tracer concentration of the tissue is always estimated from the TACs of the PET image, the arterial input function (AIF) of the model can be obtained either similarly from the TACs as an image-derived input function (IDIF) [13] or via continuous arterial blood sampling by placing a catheter in an artery in the patient’s wrist, arm, or leg for the scan period [4].

However, 1TCM does not suit for all organs. In particular, the hepatic blood flow (HBF) in human liver cannot properly be estimated with 1TCM by using a single AIF since the liver receives blood from both the hepatic artery [21] and the portal vein (PV) located in separate vessels [11]. Additionally, unlike the AIF, the PV concentration cannot be sampled noninvasively in humans and the PV is difficult to image in an accurate way with the older PET scanners due to its small size. Given the majority of the liver blood flow is from the PV [21] and, compared to the concentration in the arterial blood, the PV concentration is delayed and more dispersed because the tracer is first distributed to the intestines, spleen, pancreas, and gallbladder, the 1TCM would overestimate the arterial blood flow to the liver and misplace its peak.

Still, it is important to be able to obtain reliable information about HBF in humans. Namely, the liver is a major organ and its decreased blood flow can be an indicator of several significant health conditions. For instance, hepatic stenosis is linked with type 2 diabetes, liver cirrhosis, metabolic syndrome, and cardiovascular diseases [20]. Additionally, since PET tracers typically accumulate in the liver, the accurate modeling of liver perfusion is necessary for the development of multi-organ models.

There has been a relatively limited amount of research about HBF quantification in humans based on $^{15}$O-water PET imaging [19]. In 1999, Taniguchi et al. [23] introduced a dual-input compartment model where the blood flow from the PV was assumed to be the same as the AIF but delayed by a time coefficient estimated with the help of the spleen. A similar model was also used by Rijzewijk et al. [20], though they fitted the time delay parameter without the use of the spleen. More recently, the latter approach has also been used by Honka et al. [8] and Immonen et al. [9].

Notably, the first version of the dual-input model was introduced already 25 years ago when the spatial resolution of the PET images was considerably poorer than nowadays [14] and the total-body PET imaging of humans [12] was not yet possible. In fact, because of the size of the PV and the limited PET resolution, there has been only one publication using an IDIF for the PV [6], with the $^{11}$C-palmitate as a tracer instead of $^{15}$O-water. Noteworthily, due to the increased image resolution and the recent achievements in research on convolutional neural networks and other machine learning techniques, many automatic segmentation tools are available to be potentially utilized for finding the PV IDIF [3]. For instance, Wasserthal et al. [29] introduced a fully automatic segmentation tool TotalSegmentator for computed tomography (CT) images capable of finding 104 different anatomic structures, including liver, spleen, aorta, and hepatic PV. This raises the question whether a new version of the dual-input model with automatic segmentation and extraction of the hepatic PV could be used in HBF quantification.

In this paper, we introduce a new dual-input model using an IDIF from the aorta and the hepatic PV VOIs that can be automatically found from total-body PET/CT images with TotalSegmentator. We compare HBF estimates found with our new model to those given by three earlier modeling approaches, including the simple 1TCM, the dual-input model with the time delay estimated from the spleen as proposed by Taniguchi et al. [23], and another version of the dual-input model used by Rijzewijk et al. [20]. We systematically fit these models to the $^{15}$O-water PET data of 57 humans, after which we use statistical methods to estimate the resulting model errors in terms of the model complexity and assess whether the results are realistic. Furthermore, we estimate the differences in the HBF estimates caused by the patients’ age, sex, and weight.

**Fig. 1:**
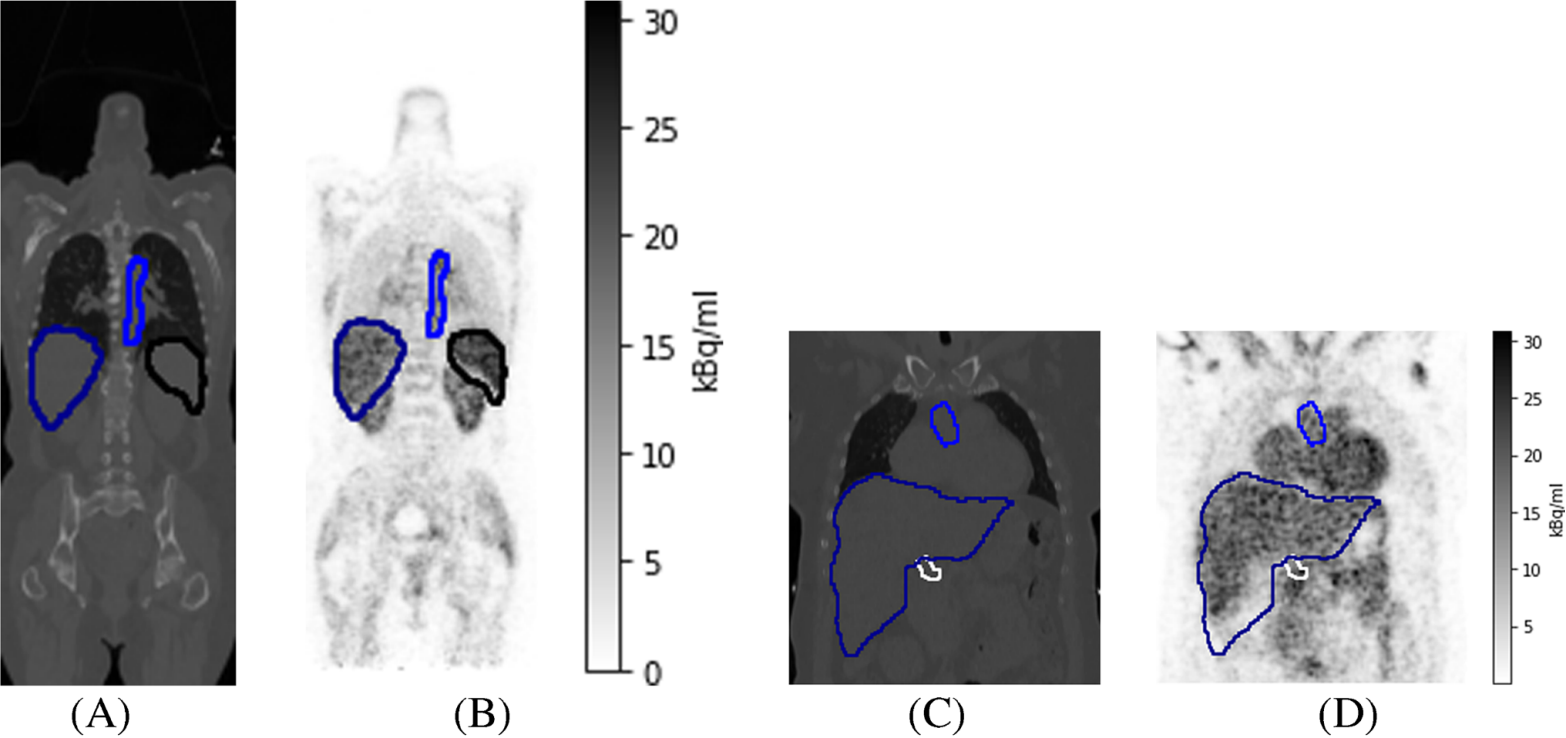
The outlines of VOIs of aorta (in blue), hepatic PV (in white), liver (in darkblue), and spleen (in black) plotted into (A) a single coronal slice of the total-body CT image, (B) the corresponding coronal slice in the time-frame between 120 and 140 seconds in the dynamic PET image, (C) a cropped image within another coronal slice of the CT, and (D) the corresponding cropped image in the same PET time-frame as in (B)

## Materials and methods

### Software

TotalSegmentator (version: 1.0) [29] was used to perform automatic segmentation of all the anatomic structures of interest in CT images. The PET and CT images and the segmentation masks were checked with Carimas (version: 2.10) [18]. Python (version: 3.9.9) [26] was used for image analysis and to extract the TACs, and R (version: 3.4.1) [24] was used for model fitting and statistical analysis.

### Data and pre-processing

The imaging data was retrospectively collected from 57 patients who had been referred for total-body PET perfusion imaging at Turku PET Centre in Turku, Finland, between August 2022 and March 2023. The patients had symptoms indicating a potential coronary artery disease, such as stable chest pain, but they had no known liver diseases. Their mean age was 64.0±8.39 years (range: 43-81 years) and the male-female sex ratio was 29:28. The patients were injected with a $^{15}$O-water dose (measured activity: 295-408 MBq) and imaged with Biograph Vision Quadra (Siemens Healthineers) PET/CT scanner while they lay at rest in a supine position. The axial field of view of Quadra is 107 cm.

The dynamic PET images consisted of $220\times 220\times 380\times 24$ image points with a voxel size of $1.65\times 1.65\times 2.80$ mm$^3$ and time intervals of 14$\,\cdot \,$5 s, 3$\,\cdot \,$10 s, 3$\,\cdot \,$20 s, and 4$\,\cdot \,$30 s. They were corrected for attenuation, randoms, decay, and scatter. The CT images had $512\times 512\times 380$ voxels with a voxel size of $0.977\times 0.977\times 2.80$ mm$^3$. Based on the segmentation of the CT images by TotalSegmentator, we chose VOIs for aorta, hepatic PV, liver, and spleen. To avoid error caused by false positive segmentation outside the anatomic structure, we only included the greatest connected component in the output by TotalSegmentator. Examples of these VOIs are shown in Fig. 1.

**Fig. 2:**
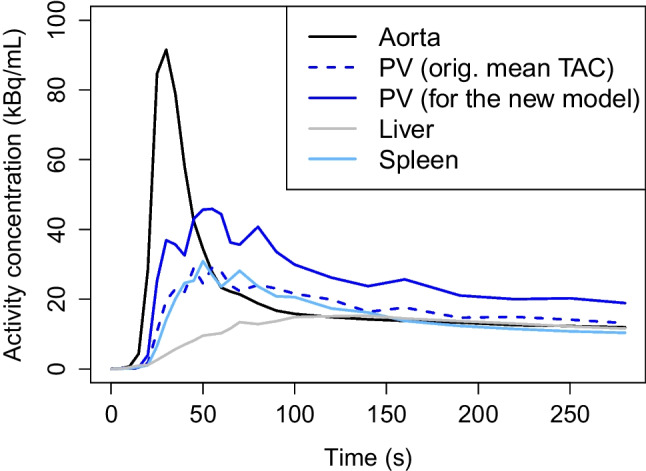
The mean TACs computed from aorta, hepatic portal vein, liver, and the spleen. For our new model, we replaced the original PV mean TAC (here in dashed blue line) by the mean TAC computed only from the hepatic PV voxels over the 90th percentile at the given time frame. All the TACs are from the same patient as the VOIs of Fig. 1

We computed mean TACs from each VOI, examples of which are shown in Fig. 2. We observed that the mean TAC of hepatic PV was quite low, possibly due to the respiratory motion during the PET scan or slight inaccuracies by TotalSegmentator. The artifact from the respiratory motion to the PV imaging has been noted earlier by Pietryga et al. [15], though they studied magnetic resonance imaging instead of dynamic PET. To avoid underestimation of the PV concentration, we replaced the original mean TAC with another mean TAC computed only from the hepatic PV voxels over the 90th percentile at the given time frame. This choice is not as sensitive to error as using the maximum TAC and instead resembles more a manual segmentation method where only the few hottest voxels are used within the area of interest. Linear interpolation was used to find TAC values at every second of the whole scan period of 280 s.

**Fig. 3:**
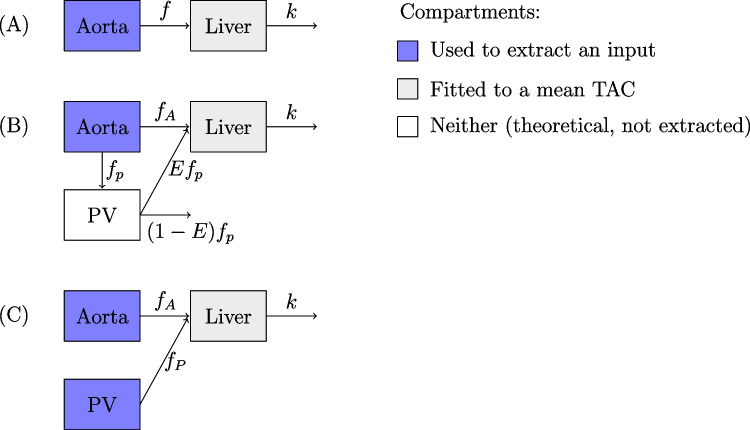
The four compartment models studied, including (A) 1TCM, (B) the dual-input model in Taniguchi et al. [23] and Rijzewijk et al. [20], and (C) our new dual-input model. We consider two variations of the dual-input model (B) that differ by the choices of time delay parameters

### Compartment models

The first and the simplest compartment model, 1TCM, requires only two functions which are $C_T(t)$ for the tracer concentration in the liver parenchyma and $C_A(t)$ for the tracer concentration in the arterial blood. The differential equation of the model is

2.1 $$\begin{aligned} \frac{\partial }{\partial t}C_T(t)=fC_A(t)-kC_T(t), \end{aligned}$$

where *f* and *k* are rate constants related to the tracer exchange from the aorta into the liver and out of the liver, respectively, as depicted in Fig. 3(A). To account for the delay caused while the tracer travels from the aorta to the liver, we use an additional delay parameter $\Delta t_1$ as in

2.2 $$\begin{aligned} \frac{\partial }{\partial t}C_T(t)=fC_A(t-\Delta t_1)-kC_T(t). \end{aligned}$$

Furthermore, while fitting the model, we do not fit the mean TAC of the liver directly to the function $C_T(t)$ because the tracer concentration of the liver is higher than the actual concentration within the tissue due to the blood within the vessels inside the liver. Instead, we fit the mean TAC to the function

2.3 $$\begin{aligned} C_{PET}(t)=(1-V_B)C_T(t)+V_BC_A(t), \end{aligned}$$

where $V_B$ is the blood volume fraction of the liver.

In the model approach by Taniguchi et al. [23], the time parameter $\Delta t_1$ is first fixed by fitting the 1TCM to the spleen as in (2.2), though with the measured tracer concentration in the spleen in place of the function $C_T(t)$. Then the actual dual-input model shown in Fig. 3(B) can be described with two differential equations:

2.4 $$\begin{aligned} \begin{aligned} \frac{\partial }{\partial t}C_{PV}(t)&=f_pC_A(t-\Delta t_1)-f_pC_{PV}(t),\\ \frac{\partial }{\partial t}C_T(t)&=f_AC_A(t-\Delta t_1-\Delta t_2)+Ef_pC_{PV}(t-\Delta t_2)-kC_T(t). \end{aligned} \end{aligned}$$

Here, the rate constants $f_A$, *k*, and $f_p$, and the portosystemic shunting parameter *E* are as Fig. 3(B), and $\Delta t_2$ is another time parameter related to the tracer exchange from aorta and PV into liver. The noteworthy detail of this dual-input model is that the tracer concentration in the portal venous system, $C_{PV}(t)$, is just a notional compartment and not extracted from any mean TAC. As can be seen in Fig. 3(B), it gives a similar input as the aorta but delayed and dispersed. All the model parameters except $\Delta t_1$ are found by fitting the mean TAC of the liver to the function

2.5 $$\begin{aligned} C_{PET}(t)=(1-V_B)C_T(t)+V_B\frac{f_AC_A(t)+Ef_pC_{PV}(t)}{f_A+Ef_p}, \end{aligned}$$

where the activity of the blood is accounted by estimating it with a weighted mean value.

The another version of this dual-input compartment model Rijzewijk et al. [20] only differs from the earlier model by Taniguchi et al. [23] by the choice of the time parameter:

2.6 $$\begin{aligned} \begin{aligned} \frac{\partial }{\partial t}C_{PV}(t)&=f_pC_A(t-\Delta t_1)-f_pC_{PV}(t),\\ \frac{\partial }{\partial t}C_T(t)&=f_AC_A(t)+Ef_pC_{PV}(t)-kC_T(t), \end{aligned} \end{aligned}$$

where the sole delay parameter $\Delta t_1$ is fixed while fitting the dual-input model instead of using the spleen. Additionally, it should be noted that, while the dual-input models in [8, 9, 20] use different names and definitions of the model parameters, they are functionally same as in (2.6). In particular, including of the shunting parameter *E* from Taniguchi et al. [23] just simplifies the parameter fitting process here. The impact of the tracer activity in the blood is again accounted by defining the function $C_{PET}(t)$ as in (2.5).

Our proposed model is quite similar to the original dual-input model. However, as shown in Fig. 3(C), we include the PV concentration measured from the hepatic PV VOI as another IDIF so the model can be described with just one differential equation

2.7 $$\begin{aligned} \frac{\partial }{\partial t}C_T(t)&=f_AC_A(t-\Delta t_1)+f_PC_{PV}(t-\Delta t_2)-kC_T(t) \end{aligned}$$

with two time delay parameters. In the model fitting, we define

2.8 $$\begin{aligned} C_{PET}(t)=(1-V_B)C_T(t)+V_B\frac{f_AC_A(t)+f_PC_{PV}(t)}{f_A+f_P}. \end{aligned}$$

The arterial HBF is obtained directly from the parameter $f_A$ and portal HBF either from the parameter $f_P$ or the product $f_P=Ef_p$ (see Fig. 3). The total HBF is $f=f_A+f_P$. We assume here that the first pass capillary extraction of water is 100%. Together with the washout-constant *k*, the total flow *f* can be used to compute the partition coefficient of water in the liver $V_T=f/k$. The functions $C_T(t)$, $C_A(t)$ and $C_{PV}(t)$ are all in the unit Bq/mL. The unit of $f_A$, $f_P$, and *f* is mL/min/mL and, if for instance $f_A=0.1$, then an average 10% of the tracer concentration from 1 mL of arterial blood is delivered into 1 mL or, equivalently, 1 cm$^3$ of liver tissue per minute. All the model parameters are positive real numbers, except the time delay parameters which have integer values corresponding with the number of seconds, and the parameters *E* and $V_B$ are also limited to the interval [0, 1].

### Model fitting

Our method of model fitting is inspired by Wang et al. [27]. Based on the differential equations (2.2), (2.4), (2.6), and (2.7), we can compute the related functions iteratively for any choices of model parameters. For instance, it follows from (2.2) that

2.9 $$\begin{aligned} C_T(i)=fC_A(i-\Delta t-1)+(1-k)C_T(i-1) \end{aligned}$$

for each time point of $i=1,2,...,280$ s. We assume here that $C_A(i)=C_T(i)=C_S(i)=C_{PV}(i)=0$ for $i=0,-1,-2,...$ . Then we define $C_{PET}(t)$ as in (2.3), (2.5), or (2.8), depending on the model. The model error is the sum of the squared differences

2.10 $$\begin{aligned} \sum ^{280}_{i=1}(\tilde{C}(i)-C_{PET}(i))^2, \end{aligned}$$

where $\tilde{C}(t)$ is the measured mean TAC from liver. To fit all the model parameters except the delay parameters, we minimize this error term (2.10) with the non-linear Newton-type minimization algorithm *nlm* in R from the initial values $f=1$, $k=1$, $f_A=0.5$, $f_p=0.6$, $E=0.9$, $f_P=0.5$, and $V_B=0.1$. Since *nlm* does not allow upper or lower bounds, we use absolute value and the inverse-logit function to restrain the range of our parameters. The time delay parameters $\Delta t_1,\Delta t_2$ are chosen by fixing them simultaneously among the possible values 0, 1, ..., 65 s based on the lowest model error.

**Table 1:** Mean ± standard deviation values of the model parameters, the partition coefficient of water $V_T$ and the ratio $f_P/f$ obtained from the 57 patients by using 1TCM, the two earlier versions of the dual-input model used by Taniguchi et al. [23] and Rijzewijk et al. [20], and our new model

Parameter	1TCM	Dual-input	Dual-input	New dual-input
		model [23]	model [20]	model
*f* (mL/min/mL)	0.622±0.239	1.236±0.422	1.254±0.379	1.229±0.612
$f_A$ (mL/min/mL)		0.176±0.154	0.210±0.086	0.299±0.168
$f_P$ (mL/min/mL)		1.060±0.355	1.044±0.332	0.930±0.520
*k* (/min)	0.621±0.255	1.381±0.418	1.390±0.365	1.603±0.720
$f_p$ (mL/min/mL)		1.227±0.347	1.277±0.657	
*E* (%)		86.5±14.4	86.7±15.2	
$V_B$ (%)	5.4±1.8	17.7±5.1	12.7±9.4	9.5±5.7
$\Delta t_1$ (s)	22.0±5.9	1.5±1.6	16.7±6.6	18.5±14.3
$\Delta t_2$ (s)		16.4±6.8		22.6±11.5
$V_T$	0.315±1.012	0.888±0.092	0.895±0.080	0.755±0.077
$f_P/f$ (%)		86.4±10.0	83.1±5.1	74.3±11.5

### Evaluation and statistical testing

After the model fitting, we compute mean relative error (MRE), mean squared error (MSE), and the Akaike’s information criteria (AIC) as in

2.11 $$\begin{aligned} \begin{aligned} \textrm{MRE}&=\frac{1}{24}\sum ^{24}_{i=1}\frac{|\tilde{C}(t_i)-C_{PET}(t_i)|}{\tilde{C}(t_i)},\quad \textrm{MSE}=\frac{1}{24}\sum ^{24}_{i=1}(\tilde{C}(t_i)-C_{PET}(t_i))^2,\\ \textrm{AIC}&=24\log (\textrm{MSE})+2N, \end{aligned} \end{aligned}$$

where $t_i$ for $i=1,...,24$ correspond with the 24 original time frames before linear interpolation and *N* is the total number of parameters (4 for 1TCM, 7 for the dual-input model by Taniguchi et al. [23], 6 for the second dual-input model without spleen, and 6 for our new model). MSE is computed between curves with unit kBq/mL. To study whether there are significant differences between these error terms between the models, we use Wilcoxon signed-rank test. We then compare the HBF estimates from our new model between women and men with the Mann-Whitney U test. Wilcoxon signed-rank test and Mann-Whitney U test are used here because they are non-parametric tests and therefore not as sensitive to potential outliers as t-tests. For all tests, we use 5% as the level of significance. We also compute Pearson’s correlation coefficients between the arterial and portal HBF and the total HBF and the patients’ age, weight, and body mass index (BMI). To test whether these correlation coefficients are statistically significant, we use the correlation test based on the t-distribution.

## Results

The estimated model parameters are summarized in Table 1. We see that the arterial HBF estimate $f_A$, portal HBF estimate $f_P$, and total HBF estimate *f* are very similar for all three dual-input models, though our new model gives a smaller value for the ratio $f_P/f$ expressing how much of the total HBF comes from the PV. In particular, the differences between these three models meant for liver are minor when compared to 1TCM, which clearly underestimates the HBF.

**Fig. 4:**
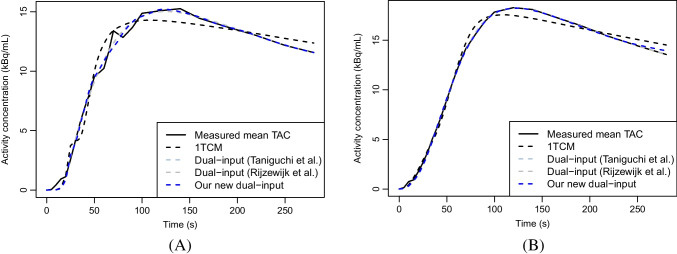
(A) The measured mean TAC of the liver VOI of a single patient and the corresponding fitted model curves given by the original dual-input proposed by Taniguchi et al. [23], the modified dual-input used by Rijzewijk et al. [20], and our new model, and (B) the same curves computed as the mean value curves over all the 57 patients. The curves not properly visible are below each other

Table 2 contains the medians of MRE, MSE, and AIC for different models. Our proposed model performed the best in terms of MRE and, out of the three dual-input models, it and the dual-input model in Rijzewijk et al. [20] also produced the smallest median MSE and AIC. According to the Wilcoxon signed-rank tests, most of the differences caused by our new model also performing better than the other models are also very clearly statistically significant (p-values$\le$0.001). The fitted model curves are shown in Fig. 4, which shows that, unlike 1TCM, all the three dual-input models produce a visually very similar fitted curve.

**Table 2:** Median values of MRE, MSE, and AIC over the 57 patients

Model	MRE (%)	MSE	AIC
1TCM	25.8***	1.14***	**7.34**
Dual-input model [23]	17.4***	0.108	14.0***
Dual-input model [20]	17.4***	**0.107**	12.0
New dual-input model	**14.2**	**0.107**	12.0

The smallest values of each column are in bold. The significantly greater error terms than those of the newly proposed model according to Wilcoxon signed-rank tests are denoted by the symbol * if p-value$\le$0.05, ** if p-value$\le$0.01, and *** if p-value$\le$0.001

**Fig. 5:**
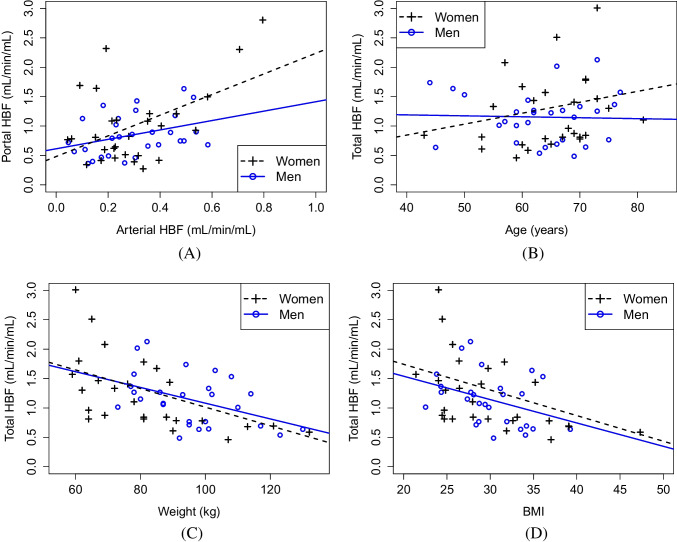
(A) The portal HBF against the arterial HBF and the total HBF against (B) age, (C) weight, and (D) BMI of the patients, when all the HBF estimates are computed with our new model. The least squares regression lines were found separately for men and women. The related Pearson’s correlation coefficients are in Table 4

Our newest model was used to study the potential sex-based differences in the HBF and the association between HBF and age, weight, and BMI. As shown in Table 3, women had higher portal HBF and lower arterial and total HBF than men but not significantly so according to the Mann-Whitney U tests. Still, as can be seen from Pearson’s correlation coefficients presented in Table 4, there is moderate positive correlation between the arterial and portal HBFs and moderate negative correlation between the total HBF and the weight or the BMI, similar both among the male and the female patients, but there is no statistically significant correlation between the total HBF and the age. This association between the variables is also visualized in Fig. 5.

**Table 3:** Median HBF estimates (mL/min/mL) from our new model over either all the 57 patients or only the 28 women or the 29 men and the p-values of the Mann-Whitney U test for sex-based differences in these estimates

HBF	All the patients	Only women	Only men	p-value
Arterial HBF	0.266	0.249	0.292	0.745
Portal HBF	0.809	0.817	0.793	0.722
Total HBF	1.15	1.03	1.22	0.663

## Discussion

In this article, we introduced a new compartment model for HBF quantification from dynamic $^{15}$O-water PET images. According to earlier research on this topic, our results from all the three dual-input models meant for HBF quantification seem realistic: We obtained a mean total HBFs varying from 1.236±0.422 to 1.229±0.612 mL/min/mL with these three models. In comparision, Taniguchi et al. [23] obtained a mean total HBF of 1.208-1.293 mL/min/g for their 88 patients depending on the liver segment and Rijzewijk et al. [20] a mean total HBF of 0.850±0.047 mL/min/mL for 18 healthy controls. In other study, Taniguchi et al. [22] reported a mean total HBF of 1.00 mL/min/g for five human patients with normal liver. Additionally, the mean percentage of the portal HBF from the total HBF was 74.3±11.5 % for our new model, which is very close to the estimate of 75% reported in Sureka et al. [21], for instance. The partition coefficient of water in these three models varied around 0.75-0.90, resembling spleen-blood partition coefficient of water in Taniguchi et al. [23], for instance.

On the other hand, based on our results, it is clear that 1TCM is ill-suited for HBF quantification. While it had the smallest median AIC due to the smallest number of parameters, its results are not useful: This model severely underestimates both HBF and the partition coefficient of water and cannot be used to estimate the arterial and portal HBFs separately.

**Table 4:** Pearson’s correlation coefficients between the specified variables computed by using the HBF estimates from our new model over either all the 57 patients or only the 28 women or the 29 men

Variables	All the patients	Only women	Only men
Arterial HBF and portal HBF	0.438***	0.499**	0.351**
Total HBF and age	0.111	0.197	-0.0329
Total HBF and weight	-0.431**	-0.403*	-0.448*
Total HBF and BMI	-0.351**	-0.348	-0.367

The p-values of correlation tests are denoted as in Table 2

Out of the three liver models, our proposed model performed the best in terms of the MRE in a statistically significant way (p-values$\le$0.001). Given there are only six model parameters in our new model as opposed to the seven of the original dual-input model, our model is also slightly more computationally efficient. While it requires extracting TACs from both aorta and hepatic PV VOIs, our result show that this does not actually require more annotation work for physicians but instead robust automatic segmentation by TotalSegmentator is accurate enough to be utilized here.

**Fig. 6:**
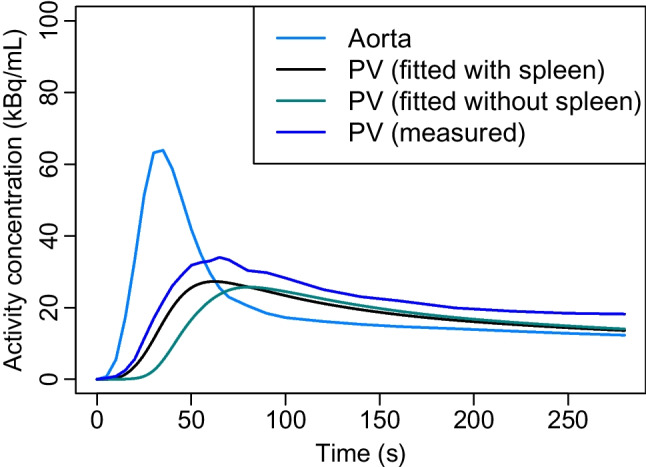
The mean input functions of our four models, including the AIF computed from the aorta, the PV input fitted with help of the spleen as proposed in Taniguchi et al. [23], the PV input fitted as in Rijzewijk et al. [20], and the PV input of our new model obtained from the mean TAC as the voxels over the 90th percentile in the PV VOI. The mean inputs are computed over all the 57 patients

As the most important difference between the three liver models is the input from the compartment used to estimate the PV concentration, we illustrated these inputs in Fig. 6. As can be seen, they are all notably lower, dispersed and delayed in time when compared to the AIF but the difference between the PV inputs is very minor. However, when comparing the tails of these inputs, it can be seen that the extracted input of our new model is slightly higher. Given we computed it as the mean TAC of the voxels over the 90th percentile separately for each time frame, this is expected if not necessary ideal: In future research, it might be worth considering if this choice could be altered. It should be considered that the PV is very small in size even compared to the higher resolution of Quadra: The mean PV diameter has been reported to be 10.9±1.9 mm in males and 10.0±1.5 mm in females [5] whereas the full width at half maximum of Quadra is about 3.5 mm [17]. Due to the small size of PV, its tracer concentration is difficult to capture fully and underestimation of the PV concentration would leave overestimation of the portal HBF. More research would be beneficial on the PV input obtained from the TotalSegmentator VOI as opposed the PV from manual segmentation and whether there is any bias in the final HBF estimates that could be avoided with partial volume effect corrections, for instance.

While there have been observed sex- and age-based differences in blood flow of organs other than liver [1, 16], we did not find any differences in the HBF estimates between men and women and only very weak positive correlation between the total HBF and the age of the female patients. However, it is possible that our study population was too old to show this relationship: Our youngest patient was 43 so more research would be needed to verify whether there are significant differences between children or adolescents and middle-aged people.

We also noted that there was moderate negative correlation between the total HBF and the weight or the BMI of the patients. This might be connected to the fact that the liver volume strongly depends of the weight of the patient. For instance, these differences in liver volumes might affect the size of different regions of the liver with respect to each other and their perfusion is not necessarily fully homogeneous despite the assumptions of the compartment model. Another factor that might cause this trend is that the potential underestimation of the PV input in the small patients with smaller PVs might lead to the overestimation of HBF of these patients. Additionally, the negative trend between HBF and weight might also be caused by certain conditions more likely in patients with higher BMI. For instance, Rijzewijk et al. [20] reported that the type 2 diabetes-high patients have lower HBF than the type 2 diabetes-low patients and both groups of diabetics have lower HBF than healthy controls. Given Rijzewijk et al. used patient groups matched in terms of BMI, their results cannot be explained by the weight of patients, but some of our patients with higher BMI possibly had type 2 diabetes or other similar underlying condition causing the decreased HBF.

## Conclusion

In this study, we introduced a new dual-input compartment model for HBF quantification from the dynamic PET images and compared it to three existing modeling approaches by systematically fitting all these models to the $^{15}$O-water PET data of 57 patients. We utilized the automatic segmentation tool TotalSegmentator to locate the necessary VOIs. We obtained realistic and quite similar HBF estimates with our new model and two other versions of the dual-input model. According to our new model, the mean arterial HBF was 0.299±0.168 mL/min/mL, the mean portal HBF 0.930±0.520 mL/min/mL, and the total HBF 1.299±0.612 mL/min/mL. Out of the three dual-input models, our proposed model performed the best in terms of MRE. We did not observe significant age- or sex-based differences in the HBF estimates but there was moderate positive correlation between the arterial and portal HBFs and negative correlation between the total HBF and the weight among both the male and the female patients.

## Data Availability

The patient data is not available due to privacy restrictions.

## Code Availability

Available at github.com/rklen/Hepatic_blood_flow
